# Longitudinal Community-Based Study of QT Interval and Mortality in Southeast Asians

**DOI:** 10.1371/journal.pone.0154901

**Published:** 2016-05-05

**Authors:** Jonathan Yap, Ai Zhen Jin, Shwe Zin Nyunt, Tze Pin Ng, A. Mark Richards, Carolyn S. P. Lam

**Affiliations:** 1 Department of Cardiology, National Heart Centre Singapore, Singapore, Singapore; 2 National Registry of Diseases Office, Health Promotion Board, Singapore, Singapore; 3 National University of Singapore, Singapore, Singapore; 4 Department of Cardiology, National University Heart Centre, Singapore, Singapore; 5 Cardiovascular Research Institute, National University Health System, Singapore, Singapore; 6 Christchurch Heart Institute, University of Otago, Christchurch, New Zealand; 7 Duke-NUS Graduate Medical School, Singapore, Singapore; Osaka University Graduate School of Medicine, JAPAN

## Abstract

**Introduction:**

The prognostic impact of QT interval prolongation has not been well studied in healthy Asians. We investigated the association between the QT interval with mortality and cardiovascular events in a healthy Southeast Asian population.

**Methods:**

The QT interval corrected for heart rate using the Bazett’s formula (QTc) was measured in 2536 (825 men, mean age 65.7±7.5 years) Singaporean adults free of cardiovascular disease in the population-based Singapore Longitudinal Ageing Study. Outcomes were all-cause mortality and incident cardiovascular events (cardiovascular mortality, myocardial infarction (MI) and/or stroke).

**Results:**

Over a mean 7.78 years (19695 person-years) of follow-up, there were 202 deaths (45 from cardiovascular causes), 62 cases of myocardial infarction and 64 cases of stroke. Adjusting for age, sex, and cardiovascular risk factors, QTcB prolongation remained independently associated with increased all-cause mortality (HR(per standard deviation) 1.27 (1.10–1.48), p = 0.0015), as well as increased risk of cardiovascular events (HR 1.20 (1.01–1.43), p = 0.0415) and MI/stroke (HR 1.22 (1.01–1.47), p = 0.0455), but not cardiovascular mortality alone (HR 1.05 (0.77–1.44), p = 0.7562).

**Conclusions:**

We provide the first community-based estimates of the independent association of QT prolongation with all-cause mortality and cardiovascular events in Southeast Asians.

## Introduction

In sharp contrast to the wealth of data from Western populations [1–9], there are very few longitudinal data on the clinical associates of QT prolongation in Southeast Asian populations. In fact, in a recent meta-analysis on the subject, all 23 studies involved were performed in Western cohorts [10]. Yet, ethnicity is known to play an important role in the relationship between QT interval and mortality. In the Atherosclerosis Risk In Communities (ARIC) study, there was a stronger association of QT interval prolongation with cardiovascular mortality in blacks compared to whites [11]. A similar trend for overall mortality was seen in blacks in the Duke databank [2]. Given the presence of ethnic differences among some non-Asian ethnicities and lack of longitudinal population-based data among Asians, we aimed to study the association between the QT interval and both mortality and cardiovascular outcomes in our community-based cohort of Southeast Asian adults without prior cardiovascular disease.

## Methods

### Study population

Singapore is a Southeast Asian city-state with a population of 5.5 million people, comprising a mix of ethnic groups including Chinese (74%), Malay (13%), and Indian (9%) ethnicities. The study population was derived from a whole area sample of community-living adults in the Singapore Longitudinal Aging Study (SLAS) [12], an ongoing prospective cohort study of ageing and health among adults aged 55 and above. The design of the SLAS has previously been described [12]. The study participants included were recruited from 2003–2012. The demographic and clinical data collected at baseline included a resting standard 12-lead electrocardiography (ECG). In this study, participants with a history of myocardial infarction, stroke, heart failure, valvular heart disease, atrial fibrillation, pacemaker or with an electrocardiogram (ECG) showing bundle branch block, ventricular pre-excitation, atrial fibrillation or 2^nd^/3^rd^ degree AV block were excluded. Written informed consent was obtained from each patient and the study was approved by the National University of Singapore institutional review board.

### Electrocardiography

All ECGs were recorded with the GE MAC 5500 ECG machine at 10 mm/mV calibration and speed of 25 mm/s. The ECGs were processed using the Marquette 12 SL algorithm (GE healthcare). The ECGs were reviewed by blinded trained staff and ECGs of inadequate quality or without a valid measurement of QTc (such as bundle branch blocks or AF) were excluded. The QT interval was measured as the time period between the earliest QRS onset to the end of the T wave, and corrected with the Bazett’s formula (QTcB = QT/√RR interval) [13]. Additional analyses performed using the Framingham formula (QTcF = QT + 154(1-60/heart rate)) [14] are shown in the Supporting Information (S1 Table and S2 Table).

### Outcomes

In Singapore, all deaths and cases of myocardial infarction (MI) and stroke outcomes are completely captured and accurately recorded in national computerized registries. Mortality data including date of death and cause of death were obtained from the Registry of Births and Deaths, using computerized record linkage. Similarly, data on incident myocardial infarction and stroke events were obtained from record linkage with the Singapore Myocardial Infarction Registry and the Singapore Stroke Registry respectively. Case ascertainment was completed up to 31^st^ Dec 2013. The primary outcome measures was all-cause mortality and secondary outcome measures included major adverse cardiovascular events (MACE) comprising cardiovascular mortality, MI and/or stroke. Other secondary outcomes studied included the individual components of MACE.

### Statistical Analysis

The Cox proportional hazards model was used to examine the association of QTc interval with all-cause mortality and MACE. Person-years of follow-up was estimated from the date of baseline interview and ECG at recruitment to the date of death or MACE or up to last follow up date (31^st^ Dec 2013). The strength of association was estimated by the hazards ratio (HRs) and their 95% confidence intervals (CIs) and P values. Subjects were divided into quartiles of QTc according to cut-points derived from the whole cohort and stratified by sex. Adjustment for potential confounding effect of other exposure variable on the QTc associations were performed using two sequential models: 1) adjusting for age, sex, ethnicity (Chinese/ Malay/ Indian/ Other); 2) adjusting for age, sex, ethnicity, BMI, history of diabetes, and history of hypertension. Linear trends of the associations were examined using ordinal values for the quartile QTc. Proportionality tests were checked and assumptions were not violated. All statistical analyses were performed with the SAS software, version 9.2. All statistical tests reported were two-sided and a p value <0.05 was considered significant.

## Results

The total of 2536 participants (mean age 65.7±7.5y) included 825 men, and a 92.1% majority of Chinese. Table 1 describes the clinical characteristics of the study population: 40.6% had hypertension and 13.3% had diabetes mellitus. The mean±SD QTcB was 442±50 ms in the overall cohort, 447±50 ms among women and 430±48 among men. From a mean of 7.78 years (IQR 8.07–9.55), corresponding to 19695 person-years of follow-up, a total of 202 subjects died (including 45 due to cardiovascular causes), and 62 subjects were registered with a diagnosis of incident myocardial infarction and 64 subjects with incident stroke.

**Table 1:** Baseline characteristics.

	All (n = 2536)	Women (n = 1711)	Men (n = 825)	P value
**Clinical characteristics**				
Age (SD)	65.7 (7.5)	65.2 (7.3)	66.7 (7.8)	<0.0001
BMI (SD)	23.7 (3.7)	23.9, (3.9)	23.5 (3.3)	0.010
Ethnicity				
Chinese (%)	2337 (92.1%)	1582 (92.5%)	755 (91.5%)	0.1420
Malay (%)	120 (4.7%)	81 (4.7%)	39 (4.7%)	
Indian (%)	60 (2.4%)	33 (1.9%)	27 (3.3%)	
Others (%)	19 (0.8%)	15 (0.9%)	4 (0.5%)	
Diabetes mellitus (%)	338 (13.3%)	219 (12.8%)	119 (14.4%)	0.2594
Hypertension (%)	1030 (40.6%)	699 (40.8%)	331 (40.1%)	0.7251
Smoker (%)	214 (8.4%)	41 (2.4%)	173 (21.0%)	<0.0001
**Laboratory**				
Cholesterol (SD) (mmol/L)	5.5 (0.9)	5.6 (0.9)	5.3 (0.9)	<0.0001
Hemoglobin (SD) (g/dl)	13.4 (1.3)	13.0 (1.1)	14.4 (1.4)	<0.0001
Creatinine (SD) (umol/L)	77.5 (36.5)	69.6 (31.6)	94.3 (40.3)	<0.0001
**ECG**				
HR (SD) (beats per min)	69 (11)	69 (10)	68 (11)	0.022
QRS (SD) (ms)	86 (10)	84 (10)	88 (10)	<0.0001
Mean QTcB (SD) (ms)	442 (50)	447 (50)	430 (48)	<0.0001
Median QTcB (IQR) (ms)	441 (409–474)	447 (416–479)	426 (397–462)	
Mean QTcF (SD) (ms)	406 (26)	410 (27)	399 (21)	<0.0001
Median QTcF (IQR) (ms)	406 (393–419)	410 (397–422)	397 (386–410)	
**Medications**				
ACE/ARB (%)	261 (10.3%)	169 (9.9%)	92 (11.2%)	0.3225
Betablocker (%)	500 (19.7%)	347 (20.3%)	153 (18.6%)	0.3035
Calcium channel blockers (%)	94 (3.7%)	67 (3.9%)	27 (3.3%)	0.4219

**Fig 1:**
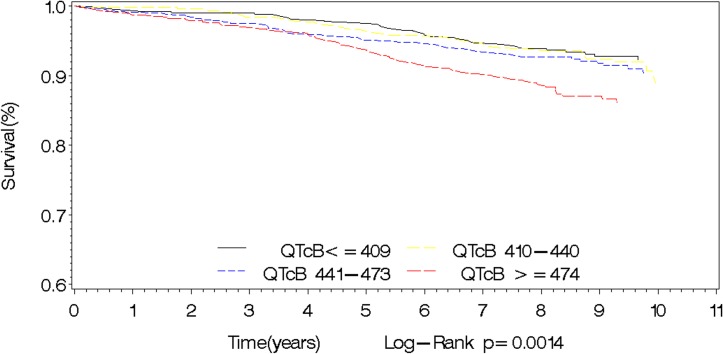
Kaplan Meier survival curves for mortality by QTcB interval.

**Fig 2:**
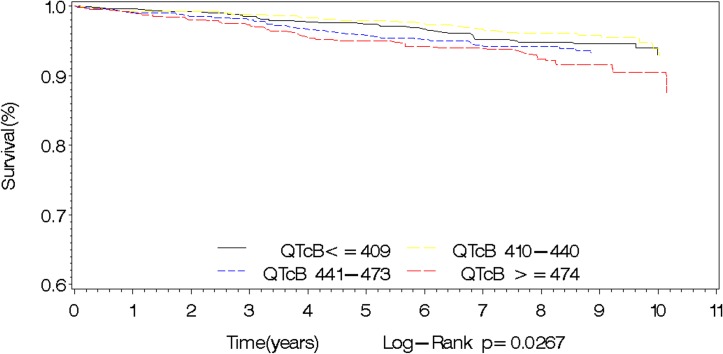
Kaplan Meier survival curves for major adverse cardiovascular outcomes by QTcB interval.

Longer QTcB interval was related to higher all-cause mortality in the overall cohort, and in men and women separately. In men, there was a significant increase in overall mortality with increasing quartiles of QTcB (P_trend_ = 0.0030). Males with QTcB≥462ms (4^th^ quartile) had the highest risk of overall mortality compared to those with QTcB≤397ms (1^st^ quartile): HR 2.48 (1.32–4.67). In women, there was a similar significant increase in overall mortality with increasing quartiles of QTcB (P_trend_ = 0.0040). Females with QTcB≥479ms (4^th^ quartile) had the highest risk of overall mortality compared to those with QTcB≤416ms (1^st^ quartile): HR 1.88 (1.15–3.09). In both men and women, there was a significant increase in overall cardiovascular events and MI/stroke with increasing quartiles of QTcB. There was no significant association with cardiovascular mortality alone in both genders (Table 2). Figs 1 and 2 show the association between the QTcB interval and overall mortality as well as MACE in the overall cohort.

**Table 2:** Sex-stratified association of QTcB (categorical) with overall and cardiovascular mortality.

		Unadjusted HR (95% CI)			
QTcB Percentiles	Range	All-cause mortality	Cardiovascular mortality/MI/Stroke	Cardiovascular mortality	MI/Stroke
**Overall**					
0–25%	≤409	1.00	1.00	1.000	1.000
25–50%	410–440	1.14 (0.74–1.75)	0.84 (0.50–1.41)	1.145 (0.486–2.697)	0.765 (0.429–1.364)
50–75%	441–473	1.24 (0.81–1.89)	1.16 (0.72–1.87)	0.959 (0.390–2.361)	1.293 (0.777–2.150)
75–100%	≥474	1.96 (1.33–2.89)	1.64 (1.05–2.57)	1.695 (0.761–3.775)	1.570 (0.958–2.572)
P_trend_		0.0005	0.0124	0.2478	0.0209
**Male**					
0–25%	≤397	1.000	1.000	1.000	1.000
25–50%	398–425	1.52 (0.77–3.01)	1.24 (0.59–2.60)	1.761 (0.421–7.369)	1.173 (0.517–2.660)
50–75%	426–461	1.90 (0.98–3.67)	1.36 (0.65–2.83)	1.118 (0.226–5.540)	1.515 (0.696–3.301)
75–100%	≥462	2.483 (1.321–4.669)	2.07 (1.04–4.09)	1.912 (0.457–7.999)	2.033 (0.965–4.283)
P_trend_		0.0030	0.0334	0.5129	0.0439
**Female**					
0–25%	≤416	1.000	1.000	1.000	1.000
25–50%	417–446	0.790 (0.435–1.435)	0.97 (0.45–2.1)	0.738 (0.234–2.327)	1.038 (0.450–2.394)
50–75%	447–478	1.127 (0.654–1.941)	1.58 (0.80–3.11)	0.905 (0.304–2.693)	1.812 (0.862–3.808)
75–100%	≥479	1.885 (1.149–3.094)	2.01 (1.04–3.86)	1.792 (0.694–4.627)	2.031 (0.973–4.239)
P_trend_		0.0040	0.0134	0.1857	0.0224

Accounting for QTcB as a continuous variable and adjusting for age, sex, ethnicity, BMI, diabetes, hypertension, cholesterol, QTcB prolongation remained independently associated with increased all-cause mortality (HR(per standard deviation) 1.27 (1.10–1.48), p = 0.0015), as well as increased risk of cardiovascular events (HR 1.20 (1.01–1.43), p = 0.0415) and MI/stroke (HR 1.22 (1.01–1.47), p = 0.0455). There was no significant association with cardiovascular mortality alone (HR 1.05 (0.77–1.44), p = 0.7562) (Table 3).

**Table 3:** Association of QTc B (continuous) with overall and cardiovascular mortality.

	Unadjusted			Model 1 *			Model 2 **		
	HR (per 10ms)	HR (per 1 SD)	P value	HR (per 10ms)	HR (per 1 SD)	P value	HR (per 10ms)	HR (per 1 SD)	P value
Overall mortality	1.052 (1.023–1.083)	1.29 (1.12–1.49)	0.0004	1.052 (1.022–1.083)	1.27 (1.10–1.47)	0.0012	1.053 (1.022–1.085)	1.28 (1.10–1.48)	0.0015
Cardiovascular mortality/MI/Stroke	1.042 (1.007–1.078)	1.23 (1.04–1.46)	0.0185	1.047 (1.011–1.085)	1.22 (1.02–1.445)	0.0282	1.045 (1.009–1.083)	1.20 (1.01–1.43)	0.0415
Cardiovascular mortality	1.028 (0.967–1.092)	1.15 (0.85–1.55)	0.3724	1.011 (0.949–1.076)	1.02 (0.74–1.39)	0.9177	1.020 (0.957–1.086)	1.05 (0.77–1.44)	0.7562
MI/Stroke	1.042 (1.004–1.082)	1.23 (1.02–1.48)	0.0281	1.050 (1.011–1.091)	1.24 (1.02–1.50)	0.0281	1.046 (1.007–1.087)	1.22 (1.01–1.47)	0.0455

*adjusted for age, sex, ethnicity

**adjusted for age, sex, ethnicity, BMI, diabetes, hypertension, cholesterol

In a sub-group analysis, 307 subjects had QTcB >500ms. Thirty passed away from all causes (3 from cardiovascular causes), 9 had myocardial infarction and 9 had stroke.

## Discussion

This study provides the first community-based data of the association of QT prolongation and risk of all-cause mortality and cardiovascular events in a general population of Southeast Asian men and women without prior cardiovascular disease.

The association of QT interval and mortality has been well described in high-risk populations. For example, Peters et al found an increased risk of mortality in patients with a prolonged QTc post myocardial infarction [3]. In patients with prior coronary artery disease [2], a prolonged QTc was found to be independently associated with overall mortality. A meta-analysis of 7 prospective cohort studies found increased risk of total or cardiovascular mortality in patients with prior cardiovascular disease [15]. The data from population-based studies however is less well-established. This same meta-analysis found that in the general population, an increased risk in mortality was likely to be small and difficult to detect reliably [15]. The original Framingham Heart Study did not find any association between the QTc and overall and cardiovascular mortality in a healthy adult population [16]. In a large Danish primary care population of more than 100,000 patients, a prolonged QTc was associated with increased all-cause and cardiovascular mortality [7], but these included patients with cardiovascular disease (albeit small <10%). Another meta-analysis of participants free of cardiovascular disease found a significant association with total and cardiovascular mortality [10]. Our study similarly found a significant association between overall mortality and QTc prolongation. A relatively limited number of cardiovascular mortality events may have limited our power to detect a significant association with cardiovascular mortality in this study, although the point estimate showed a trend towards increased cardiovascular mortality with QTc prolongation. We postulate that some of the all-cause mortality events were, in fact, unreported cardiovascular deaths (which could not be adjudicated in our national registry with the strict standards of a clinical trial). Furthermore, the intake of beta blockers in about 20% of the population may have exerted some protective effect on cardiovascular mortality.

Beyond mortality, there have been fewer studies examining the association between QTc and cardiovascular events. The REGARDS (REasons for Geographic and Racial Differences in Stroke) study found increased stroke risk with increasing QTc intervals [17] but included participants with prior cardiovascular disease. In participants initially free of cardiovascular disease the ARIC study found an increased incidence of subsequent new onset coronary heart disease in those with prolonged QT intervals [11]. More recently, the Multi-Ethnic Study of Atherosclerosis (MESA) study also found significant associations between QT interval and incident cardiovascular events (including stroke, cardiovascular disease and heart failure) in individuals free of cardiovascular disease on recruitment [8]. Consistent with these prior studies, we found that QTc interval prolongation was associated with a significant increase in MACE (comprising cardiovascular deaths, MI and stroke) as well as MI or stroke alone in our population-based cohort of Asian adults.

The vast majority of published studies have focused on Western populations. In contrast, data has been scarce among Asians: the MESA study included approximately 10% Chinese Americans [8], while the REGARDS study oversampled African Americans [17]. Ethnicity has been shown to have an impact on the relationship between the QT interval and mortality. The ARIC study found a greater increase risk in cardiovascular mortality with increasing quintiles of QTc among blacks compared to whites. Similarly, in the Duke Databank for Cardiovascular Disease, a greater increase in mortality risk with QT prolongation was observed for blacks compared to other races [12]. There has been some data published in Japanese populations. A study of 3500 elderly Japanese subjects found a significant association between QTc interval and overall mortality [18], while another Japanese study found an increased risk of stroke and coronary heart disease with QT prolongation in males [19]. In this first study on community-based Southeast Asians, we found that QT prolongation was independently related to all-cause mortality and MACE, thus extending prior data to Southeast Asians. However, the smaller numbers of Malays and Indians in this study precluded inter-ethnic comparisons among the different Asian ethnicities.

While the association of QT prolongation with mortality and MACE appears consistent across various ethnic groups, the strength of the association and QT interval cut-off for increased risk may vary from one ethnic group to another. Interestingly, the QT value defining the lower boundary of QTcB for the upper quartile of our cohort was 474ms. This is higher than corresponding cut-offs reported in Western community-based cohorts. The cut-off for the upper quintile of QTcB in the Framingham cohort was 437ms while that of the Third National Health and Nutrition Examination Survey (NHANES III) was 448ms [20]. In the NHANES III study, the 95^th^ percentile of QTcB(≥470ms) had an increased HR of mortality of 1.42 (1.03–1.94). This risk is lower than that seen in our cohort- our upper quartile of QTcB (≥474ms) had an increased HR of 1.96 (1.33–2.89). In the Rotterdam study, the upper quartile cut-offs of QTcB for men was 437ms (vs 462ms in our men) and 446ms in women (vs 479ms in our female cohort) [21]. In males, the HR for mortality for the upper quartile of 1.3 (0.7–2.4) was lower in the Rotterdam study as compared to our study (HR 2.48 (1.32–4.67)). In females, this was fairly similar (HR 2.4 (1.1–5.3) vs HR 1.89 (1.14–3.09). Prior descriptive studies have shown differences in QT intervals between different ethnic groups. Macfarlane et al reported longer QTc intervals in Chinese compared to Whites [22]. Although, these populations may not be directly comparable, our study raises questions that there may be different (higher) QT interval cut-offs amongst our Southeast Asian cohort compared to white adults and that these cut-offs may potentially attribute higher risks–this warrants prospective study.

There have been some postulates regarding the mechanism between the prolonged QTc interval and adverse cardiovascular events. A prolonged QTc may be a reflection of abnormalities in the autonomic nervous system and in ventricular repolarisation [23], leading to increased risk of ventricular arrhythmias. Intriguingly, a small study of 200 Laotian refugees showed that a prolonged QTc interval was related to seizure-like episodes in sleep with a possible correlation to thiamine deficiency (staple diet of fermented fish containing antithiamine compounds) [24] and hypokalemia [24,25]. The QT interval has also been shown to be associated with subclinical atherosclerosis and may be a marker of generalised atherosclerosis [26].

We acknowledge that the QT interval was only measured from the baseline resting ECG in our study; and that the QT interval is dynamic. This is an inherent limitation to most epidemiologic studies. We used the Bazett’s formula since this is most commonly used in prior studies and allows comparison with other studies (17 out of 23 studies in a recent meta-analysis on the impact of QTc on mortality used the Bazett’s formula). Several studies used linear regression functions for the correction of the QT interval [7,8] and our results using the Framingham formula show similar trends (S1 and S2 Tables). QTc prolonging medications have been shown to be an important contributor to prolonged QT [27], but data on these medications were not available. However, this is a population-based study of community-ambulant individuals with few comorbidities, so we do not expect that medications played a significant role in the majority of cases. We also acknowledge that prospective data on sudden cardiac death was not available. Although the QT interval has prognostic implications and may aid in risk stratification, the actual individual mortality benefit is less clear. Further studies on intervention strategies will be required.

## Conclusions

We provide the first community-based estimates of the independent association of QT prolongation with all-cause mortality and cardiovascular events in Southeast Asians. The implications of these findings for ethnicity-specific risk stratification deserves further study.

## Supporting Information

S1 Table Association of QTcF (continuous) with overall and cardiovascular mortality. (DOCX)

S2 Table Sex-stratified association of QTcF (categorical) with overall and cardiovascular mortality. (DOCX)
