# Supplementary material for: Longitudinal Community-Based Study of QT Interval and Mortality in Southeast Asians
Source: PLoS One. 2016 May 5;11(5):e0154901. doi: 10.1371/journal.pone.0154901 (PMC4858262; doi:10.1371/journal.pone.0154901)
Supplement: S1 Table — (DOCX) [file pone.0154901.s001.docx]

**S1 Table. Association of QTcF (continuous) with overall and cardiovascular mortality**

|  | Unadjusted | | | Model 1* | | | Model 2** | | |
| --- | --- | --- | --- | --- | --- | --- | --- | --- | --- |
|  | HR (per 1ms) | HR (per 1 SD) | P value | HR (per 1ms) | HR (per 1 SD) | P value | HR (per 1ms) | HR (per 1 SD) | P value |
| Overall mortality | 1.008 (1.002-1.014) | 1.22 (1.06-1.42) | 0.0074 | 1.006 (1.000-1.012) | 1.17 (1.01-1.35) | 0.0388 | 1.006 (1.000-1.012) | 1.18 (1.01-1.37) | 0.0362 |
| Cardiovascular mortality/MI/Stroke | 1.005 (0.998-1.012) | 1.14 (0.95-1.37) | 0.1576 | 1.006 (0.999-1.013) | 1.17 (0.97-1.41) | 0.0924 | 1.006 (0.998-1.013) | 1.15 (0.96-1.39) | 0.1363 |
| Cardiovascular mortality | 1.013 (1.001-1.025) | 1.39 (1.03-1.87) | 0.0302 | 1.008 (0.996-1.020) | 1.22 (0.90-1.65) | 0.1936 | 1.008 (0.997-1.019) | 1.22 (0.92-1.63) | 0.1686 |
| MI/Stroke | 1.004 (0.996-1.011) | 1.10 (0.90-1.34) | 0.3600 | 1.005 (0.997-1.013) | 1.15 (0.93-1.40) | 0.1897 | 1.005 (0.997-1.013) | 1.13 (0.91-1.39) | 0.2608 |

*adjusted for age,sex, ethnicity

**adjusted for age, sex, ethnicity, BMI, diabetes, hypertension, cholesterol
