# Supplementary material for: Longitudinal Community-Based Study of QT Interval and Mortality in Southeast Asians
Source: PLoS One. 2016 May 5;11(5):e0154901. doi: 10.1371/journal.pone.0154901 (PMC4858262; doi:10.1371/journal.pone.0154901)
Supplement: S2 Table — (DOCX) [file pone.0154901.s002.docx]

**S2 Table. Sex-stratified association of QTcF (categorical) with overall and cardiovascular mortality**

|  |  | Unadjusted HR (95% CI) | | | |
| --- | --- | --- | --- | --- | --- |
| QTcF Percentiles | Range | All-cause mortality | Cardiovascular mortality/MI/Stroke | Cardiovascular mortality | MI/Stroke |
| Overall |  |  |  |  |  |
| 0-25% | ≤393 | 1.00 | 1.00 | 1.00 | 1.00 |
| 25-50% | 394-406 | 1.15 (0.77-1.71) | 1.12 (0.69-1.82) | 1.51 (0.57-3.97) | 1.06 (0.62-1.80) |
| 50-75% | 407-418 | 1.07 (0.71-1.61) | 0.98 (0.59-1.63) | 1.88 (0.73-4.77) | 1.09 (0.64-1.85) |
| 75-100% | ≥419 | 1.39 (0.94-2.04) | 1.56 (1.00-2.46) | 2.55 (1.05-6.21) | 1.52 (0.93-2.50) |
| P- trend |  | 0.1377 | 0.0825 | 0.0296 | 0.1001 |
| Male |  |  |  |  |  |
| 0-25% | ≤386 | 1.00 | 1.00 | 1.00 | 1.00 |
| 25-50% | 387-397 | 1.21 (0.60-2.42 | 1.13 (0.53-2.40) | 1.62 (0.27-9.69) | 1.14 (0.50-2.59) |
| 50-75% | 398-409 | 1.98 (1.05-3.73) | 1.84 (0.92-3.67) | 3.51 (0.71-17.39) | 1.86 (0.88-3.93) |
| 75-100% | ≥410 | 2.45 (1.32-4.53) | 1.68 (0.83-3.40) | 3.14 (0.61-16.18) | 1.67 (0.77-3.59) |
| P- trend |  | 0.0011 | 0.0702 | 0.0990 | 0.1003 |
| Female |  |  |  |  |  |
| 0-25% | ≤397 | 1.00 | 1.00 | 1.00 | 1.00 |
| 25-50% | 398-409 | 1.34 (0.80-2.22) | 1.57 (0.79-3.10) | 2.48 (0.76-8.04) | 1.46 (0.69-3.08) |
| 50-75% | 410-421 | 0.76 (0.42-1.36) | 1.24 (0.60-2.54) | 1.35 (0.36-5.01) | 1.26 (0.58-2.73) |
| 75-100% | ≥422 | 1.43 (0.87-2.38) | 1.86 (0.96-3.62) | 3.11 (0.99-9.77) | 1.79 (0.87-3.70) |
| P- trend |  | 0.4567 | 0.1216 | 0.1099 | 0.1619 |
